# Neutron structure of human carbonic anhydrase II in complex with methazolamide: mapping the solvent and hydrogen-bonding patterns of an effective clinical drug

**DOI:** 10.1107/S2052252516010514

**Published:** 2016-07-22

**Authors:** Mayank Aggarwal, Andrey Y. Kovalevsky, Hector Velazquez, S. Zoë Fisher, Jeremy C. Smith, Robert McKenna

**Affiliations:** a Biology and Soft Matter Division, Oak Ridge National Laboratory, Oak Ridge, TN 37831, USA; b Center for Molecular Biophysics, Oak Ridge National Laboratory, Oak Ridge, TN 37831, USA; c Department of Biochemistry, Cellular and Molecular Biology, University of Tennessee, Knoxville, TN 37996, USA; d Scientific Activities Division, European Spallation Source, 22100 Lund, Sweden; e Department of Biochemistry and Molecular Biology, College of Medicine, University of Florida, Gainesville, FL 32610, USA

**Keywords:** human carbonic anhydrase, acetazolamide, methazolamide, neutron structure, drug binding

## Abstract

Carbonic anhydrases (CAs; EC 4.2.1.1) catalyze the interconversion of CO_2_ and HCO_3_^−^, and their inhibitors have long been used as diuretics and as a therapeutic treatment for many disorders such as glaucoma and epilepsy. Acetazolamide (AZM) and methazolamide (MZM, a methyl derivative of AZM) are two of the classical CA inhibitory drugs that have been used clinically for decades. The jointly refined X-ray/neutron structure of MZM in complex with human CA isoform II (hCA II) has been determined to a resolution of 2.2 Å with an *R*_cryst_ of ∼16.0%. Presented in this article, along with only the second neutron structure of a clinical drug-bound hCA, is an in-depth structural comparison and analyses of differences in hydrogen-bonding network, water-molecule orientation and solvent displacement that take place upon the binding of AZM and MZM in the active site of hCA II. Even though MZM is slightly more hydrophobic and displaces more waters than AZM, the overall binding affinity (*K*_i_) for both of the drugs against hCA II is similar (∼10 n*M*). The plausible reasons behind this finding have also been discussed using molecular dynamics and X-ray crystal structures of hCA II–MZM determined at cryotemperature and room temperature. This study not only allows a direct comparison of the hydrogen bonding, protonation states and solvent orientation/displacement of AZM and MZM, but also shows the significant effect that the methyl derivative has on the solvent organization in the hCA II active site.

## Abstract 2

The room-temperature X-ray/neutron structure of human carbonic anhydrase II in complex with methazolamide has been determined. The study provides new insights into the hydrogen-bonding changes that take place within the active site of an enzyme upon drug binding, including changes in the orientation of the H atoms of residues and waters, and the observed displacement of water.

## 1. Introduction   

Carbonic anhydrases (CAs) are zinc metalloenzymes that are involved in a wide range of physiological functions from pH regulation to the transport of CO_2_. CA inhibitors (CAIs) are currently used in the treatment of glaucoma, high blood pressure, epilepsy, altitude sickness, gastric and duodenal ulcers, neurological disorders and osteoporosis (Aggarwal *et al.*, 2013 ▸; Supuran, 2008 ▸). Human CA II (hCA II) is one of 12 catalytically active isoforms, with a *k*_cat_/*K*_m_ of 1.6 × 10^8^ *M* ^−1^ s^−1^, and is ubiquitously expressed in almost all tissue types (Frost & McKenna, 2013 ▸; Supuran, 2008 ▸).

Classical CAIs utilize a sulfonamide group, *R*SO_2_NH_2_, as their primary zinc-binding group (ZBG) and have been in clinical use for more than 50 years as diuretics and antiglaucoma drugs. Today there are at least 20 drugs against CAs that are in clinical use. In addition to the established use of CAIs, they are also being developed for the treatment of obesity, cancer and pain (Supuran, 2008 ▸; Aggarwal *et al.*, 2013 ▸). However, the hCAs share a range of sequence identity (from 25 to 70% at the amino-acid level) and as such there is substantial off-target binding to other isoforms, reducing drug effectiveness and causing side effects. Hence, there is a need to design efficient, hCA isoform-specific drugs (Supuran, 2008 ▸; Alterio *et al.*, 2009 ▸; Aggarwal *et al.*, 2013 ▸).

**Figure 1:**
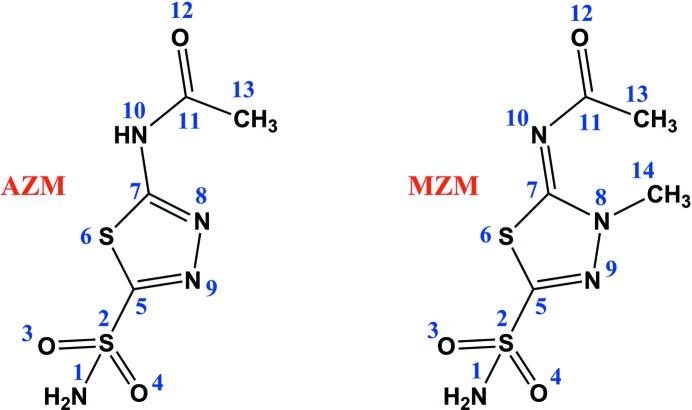
Structural formulae and numbering of AZM and MZM.

Methazolamide {MZM; *N*-[5-(aminosulfonyl)-3-methyl-1,3,4-thiadiazol-2(3*H*)-ylidene]acetamide} is a clinically used, orally administered CAI marketed under the trade name Neptazene. MZM is a methyl derivative of another classical CAI, acetazolamide (AZM, marketed as Diamox; Maren *et al.*, 1993 ▸); Fig. 1 ▸ shows the structural formulae of AZM and MZM. Both are only weakly soluble in water, but are readily soluble in organic solvents such as dimethyl sulfoxide. The solubilities in water of AZM and MZM are 2.8 and 1.7 mg ml^−1^, respectively (Yalkowsky & Dannenfelser, 1992 ▸). MZM was designed based on AZM to decrease its ionization so that its intraocular penetration was enhanced in the treatment of glaucoma. This strategy was successful and MZM became the preferred drug over AZM for systemic administration owing to various factors, including greater stability, longer half-life, lower dose requirement and fewer side effects (Bartlett & Holdeman, 2008 ▸). Despite MZM having desirable properties from a commercial perspective, both drugs have similar hCA II inhibition constants of ∼10 n*M* (Maren *et al.*, 1993 ▸).

Over 400 X-ray crystal structures of hCA II have been deposited in the Protein Data Bank (PDB) and about half of these are in complex with inhibitors (Aggarwal *et al.*, 2013 ▸). In contrast, there are five neutron crystal structures, of which only one is in complex with an inhibitor (AZM); the other four are pH studies on uncomplexed hCA II (Fisher *et al.*, 2010 ▸, 2011 ▸, 2012 ▸; Michalczyk *et al.*, 2015 ▸). For X-ray crystallography, diffraction resolution is a limiting factor in structure-based drug design owing to difficulties in placing hydrogen (H) atoms. H atoms make up ∼50% of the atoms in a protein. Currently, neutron crystallography is the only direct visualiz­ation method to observe H or its isotope deuterium (D) in protein crystal structures (Niimura & Bau, 2008 ▸; Niimura & Podjarny, 2011 ▸). A recent study of X-ray and neutron crystal structures revealed that at atomic or subatomic resolution (<1 Å) only a small fraction of H atoms can be observed in X-ray structures and usually only in well ordered areas. In contrast, neutron crystal structures can reveal H/D atoms with a high level of certainty at medium (2.0–2.5 Å) resolution (Gardberg *et al.*, 2010 ▸). Despite the obvious advantage of obtaining a neutron crystal structure, there are still only a few examples of clinical drugs bound to enzymes, such as dihydro­folate reductase (DHFR) with methotrexate (Bennett *et al.*, 2006 ▸), human immunodeficiency virus (HIV) protease with amprenavir (Weber *et al.*, 2013 ▸), and hCA II with AZM (Fisher *et al.*, 2012 ▸).

## 2. Experimental procedures   

### 2.1. Protein expression and purification   

The recombinant gene for human carbonic anhydrase isoform II (hCA II) was cloned into a pET-32b plasmid vector with an ampicillin-resistance gene and expressed in *Escherichia coli* BL21 (DE3) cells as described previously (Aggarwal *et al.*, 2014 ▸). The culture was grown at 37°C in the presence of ampicillin (100 mg ml^−1^) until it reached an OD_600_ of 0.6, and was thereafter induced with IPTG (100 mg ml^−1^) for protein expression. The lysate was then purified by affinity chromatography using a *p*-aminomethylbenzenesulfonamide column. Nonspecifically bound proteins were washed off the column using wash buffer (200 m*M* sodium sulfate, 100 m*M* Tris) at pH 7.0 and pH 9.0, finally eluting the protein with elution buffer (400 m*M* sodium azide). The enzyme was thereafter buffer-exchanged with 50 m*M* Tris pH 7.8 to remove sodium azide and concentrated to 10 mg ml^−1^ using a 10 kDa filter.

### 2.2. Sample preparation   

Drops of 200 µl (100 µl protein solution and 100 µl precipitant solution) were equilibrated against precipitant solution (1.6 *M* sodium citrate, 50 m*M* Tris–HCl pH 8.0) at room temperature (298 K), and crystals were observed after five weeks. The crystals were then soaked overnight with ∼1 m*M* methazolamide. Based on visual inspection, a single large crystal (∼0.7 mm^3^) was mounted in a quartz capillary containing the precipitant solution made in D_2_O. The labile H atoms were allowed to exchange with deuterium by vapor diffusion for four weeks before starting data collection.

### 2.3. Data collection   

X-ray data were collected in-house using an R-AXIS IV^++^ image-plate system on a Rigaku MicroMax 007 HF Cu rotating-anode generator operating at 40 kV and 30 mA with Osmic VariMax HR optics. The crystal-to-detector distance was set to 100 mm and data were collected with oscillation steps of 1° (with an exposure time of 30 s) per image. The neutron crystal diffraction data were collected on the IMAGINE instrument, with a 20 h exposure time and images collected every 7°.

### 2.4. Data processing and refinement   

X-ray diffraction data indexing, integration and scaling were performed using *HKL*-3000 (Minor *et al.*, 2006 ▸). Starting phases were calculated from PDB entry 3ks3 (Avvaru *et al.*, 2010 ▸) with waters removed. Refinement using the *PHENIX* package (Adams *et al.*, 2010 ▸), with 5% of the unique reflections selected randomly and excluded from the refinement data set for the purpose of *R*_free_ calculations (Brünger, 1992 ▸), was alternated with manual refitting of the model in *Coot* (Emsley & Cowtan, 2004 ▸). The validity of the final model was assessed by *PROCHECK* (Laskowski *et al.*, 1993 ▸). The neutron data were processed using the Daresbury Laboratory *LAUE* suite program *LAUEGEN* (Campbell, 1995 ▸) modified to account for the cylindrical geometry of the detector (Campbell *et al.*, 1998 ▸). *LSCALE* (Arzt *et al.*, 1999 ▸) was used to determine the wavelength-normalization curve using the intensities of symmetry-equivalent reflections measured at different wavelengths. No explicit absorption corrections were applied. These data were then merged in *SCALA* (Weiss, 2001 ▸).

### 2.5. Joint X-ray/neutron (XN) structure refinement   

The joint XN structure of hCA II–MZM was determined using *nCNS* (Adams *et al.*, 2009 ▸) and manipulated in *Coot* (Emsley & Cowtan, 2004 ▸). After initial rigid-body refinement, several cycles of positional, atomic displacement parameter and occupancy refinement followed. Between each cycle the structure was checked, side-chain conformations were altered and water-molecule orientations were built based on the *F*_o_ − *F*_c_ difference neutron scattering-length density map. The 2*F*_o_ − *F*_c_ and *F*_o_ − *F*_c_ neutron scattering-length density maps were then examined to determine the correct orientation of hydroxyl groups and the protonation states of His and Lys residues. The protonation states of some disordered side chains could not be obtained directly and remained ambiguous. All water molecules were refined as D_2_O. Initially, water O atoms were positioned according to their electron-density peaks, and then were shifted slightly in accordance with the neutron scattering-length density maps. Labile H positions in hCA II were modeled as D atoms and the occupancy of the D atoms was then allowed to refine within the range −0.56 to 1.00 (the scattering length of H is −0.56 times the scattering length of D). Before depositing the final structure in the PDB, a script was run that converts a record for the coordinate of D atom into two records corresponding to an H and a D atom partially occupying the same site, both with positive partial occupancies that add up to unity.

### 2.6. Solvation-energy calculations   

The nonpolar contributions to the solvation free energies of the free ligands were estimated through the use of surface-area calculations (Kollman *et al.*, 2000 ▸) as implemented in the *sander* module of *AMBER* 12 (Case *et al.*, 2012 ▸). The surface-area dependent contributions to solvation of the free ligand *x* were estimated as
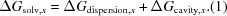
Short molecular-dynamics (MD) simulations were performed on the AZM and MZM ligands in aqueous solution. Charges for the AZM and MZM ligands were derived from quantum-mechanical calculations at the HF level of theory with the 6-31G* basis set after the geometries had been optimized with B3LYP and the same basis set. A brief MD run of 1000 MD steps was performed to heat the system to 300 K. Once the system reached 300 K, 50 000 MD steps were used to ensure equilibration of the system at 300 K. A 2 ns trajectory was subsequently collected for each complex with a 2 fs timestep. A snapshot from the trajectory was saved every five MD steps, but only the first 1000 snapshots are used for further analysis. Fig. 4 shows the distribution of solvation free energies calculated from the MD snapshots.

## 3. Results and discussion   

### 3.1. hCA II–MZM structure   

**Figure 2:**
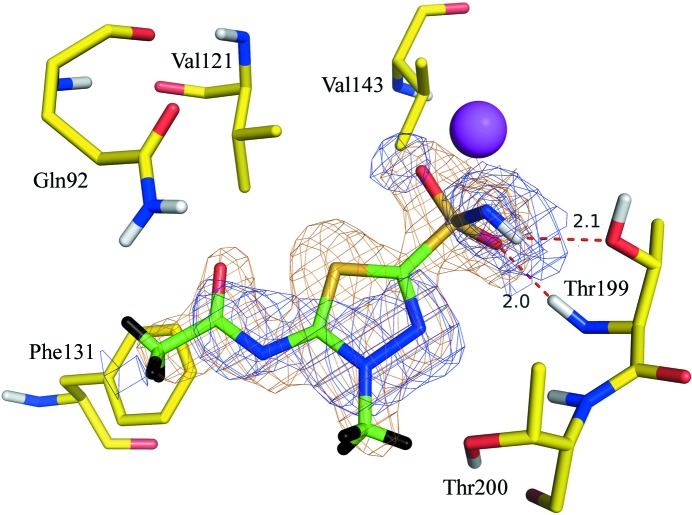
Stick representation of MZM (green) bound in the active site of hCA II (yellow). 2*F*_o_ − *F*_c_ X-ray (orange mesh) and neutron (blue mesh) maps are contoured at 1.2σ. The zinc ion is represented by a magenta sphere and hydrogen bonds are depicted as red dashes. Unexchanged nonpolar H and exchanged polar D atoms are colored black and white, respectively.

This study describes the second neutron structure of a complex of hCA II, that with MZM (Fig. 2 ▸), and for the first time allows a detailed neutron-based structural comparison of hCA II bound to two clinically used drugs. This presents an opportunity for analysis of the drug protonation state, water-molecule orientation and displacement, and hydrogen-bonding patterns. The analysis provides a unique chance to compare binding interactions and correlate them with measured inhibition constants, and may provide valuable information for the development of CA II-selective and CA isoform-selective inhibitors.

The neutron crystal diffraction data were collected from an H/D-exchanged single crystal (∼0.7 mm^3^) of hCA II co-crystallized with MZM in a 1:2 molar ratio to 2.2 Å resolution using the IMAGINE instrument at Oak Ridge National Laboratory. For the joint refinement, room-temperature (RT) X-ray crystal diffraction data were collected in-house on a Rigaku HomeFlux system to 1.5 Å resolution. Complete crystallographic and structure-refinement statistics for the diffraction data and refinement are given in Table 1 ▸.

**Figure 3:**
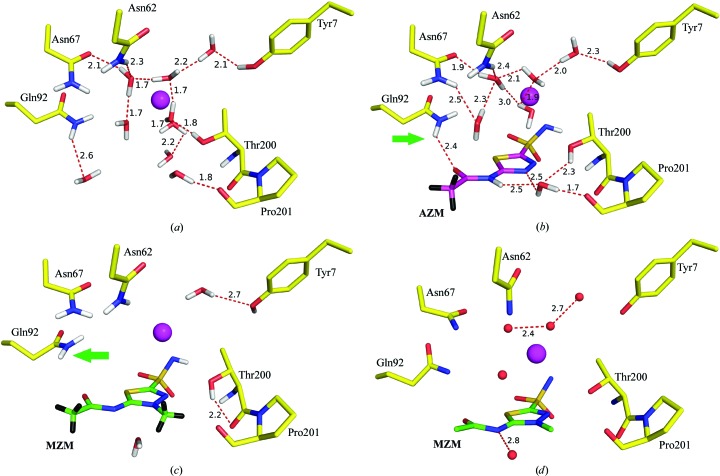
Active site at pH 7.8 of (*a*) unbound hCA II (Fisher *et al.*, 2011 ▸), (*b*) hCA II in complex with AZM (Fisher *et al.*, 2012 ▸) and (*c*) hCA II in complex with MZM, showing differences in hydrogen-bonding patterns as observed by neutron diffraction at RT. (*d*) MZM bound in the active site of hCA II as observed by X-ray diffraction at 100 K. MZM binding displaces four water molecules. Change in the orientation of Gln92 (green arrows) also causes a loss of a hydrogen bond to the inhibitor.

OMIT (*F*_o_ − *F*_c_) X-ray electron density corresponding to MZM was clearly seen in the active site of hCA II. A molecule of MZM was then modeled into the difference map and subsequently refined. Shown in Fig. 2 ▸ are the 2*F*_o_ − *F*_c_ electron and neutron scattering-length density maps of MZM. As expected, based on the previously reported AZM complex study (Fisher *et al.*, 2012 ▸), the MZM sulfonamide (ZBG) was also deprotonated. Surprisingly, MZM displaced seven of the nine water molecules observed in the unbound active site. Three of these waters were also reported to be displaced in the AZM complex structure and superpose onto N1, O3 and O12 of AZM/MZM (Fig. 3 ▸; for numbering refer to Fig. 1 ▸).

### 3.2. Comparative analysis of hCA II–AZM and hCA II–MZM   

In the AZM complex, a water molecule bridges hydrogen bonds from the side chain of Thr200 and the main chain of Pro201 to the endocyclic N8 atom and the amide N10 atom of AZM (Figs. 3 ▸ *a* and 3 ▸ *b*), whereas in the MZM complex the additional hydrophobic methyl group (C14, located in this region) expels this water and orients the H atoms away from the inhibitor, thereby precluding any possibility of hydrogen bonding. However, in all three structures the threonine still satisfies a hydrogen bond and hence the inhibitor binding creates a structural perturbation that induces a conformational change to different energy minima (Fig. 3 ▸ *c*).

Interestingly, the RT neutron/X-ray structure of MZM displaced seven of the nine water molecules compared with the RT neutron/X-ray structure of the unbound active site, and four more waters than the RT neutron/X-ray structure of the hCA II–AZM complex. This suggests that the addition of the methyl group to MZM, when compared with AZM, disrupts and displaces significantly more ordered solvent.

However, an equivalent cryo X-ray structure of the hCA II–MZM complex showed the presence of these waters (Fig. 3 ▸ *d*). Hence, the low temperature of the cryo structure was sufficient to cause a reduction in motion (free energy) and the ‘freezing in’ of these waters, distorting the structural view under the more physiological (RT) conditions. It is well known that ligand binding within enzyme active sites displaces water molecules, and this event is an important factor in modulating the binding affinity of a ligand. This ‘freeing’ expulsion of waters comes at an energetic expense involving the breaking of the hydrogen bonds that the displaced waters previously formed. In addition, it also adds to the overall entropy of the system by releasing the waters into the bulk solvent, thereby reducing the Gibbs free energy and enhancing the binding affinity of the ligand. In the case of AZM binding to hCA II at RT, the water molecules form an elaborate hydrogen-bonding network (Figs. 3 ▸ *a* and 3 ▸ *b*). In contrast, the addition of a methyl group in MZM displaces/destabilizes four additional waters in the active site, which should theoretically enhance the binding affinity of MZM (Figs. 3 ▸ *a* and 3 ▸ *c*). However, the entropy increase comes at the expense of a loss of hydrogen bonds, which will enthalpically compensate for the entropic gain. Also of note is the change in orientation of the side chain of Gln92, which positions its side-chain amide group such that the Gln92 N–Gln92 D–MZM O12 angle approaches 90° (green arrow in Fig. 3 ▸ *b*), essentially reducing any stability gain otherwise observed in the hCA II–AZM structure (Fig. 3 ▸ *b*).

### 3.3. Solvation-energy calculations   

Equally important in determining binding equilibria in aqueous solutions are the solvation free energies of the free ligands. The relationship of structure to the thermodynamics of ligand binding, including entropy, enthalpy compensation and the need for MD to capture fleeting protein–ligand interactions, has previously been elaborated by Bradbrook and coworkers for concanavalin A (Bradbrook *et al.*, 1998 ▸). To determine how these may counteract the effects of active-site water-molecule displacement, an *in silico* approach was taken and Δ*G*_solv_ (the volume-dependent component of the solvation energies of the free ligands) was calculated. Here, Δ*G*_solv_ consists of a sum of two terms (Tan *et al.*, 2007 ▸): Δ*G*_dispersion_ for attractive dispersive interactions and Δ*G*_cavity_, a repulsive cavity term that represents the free-energy penalty that must be paid in order to expel water molecules for solvation. The Δ*G*_dispersion_ and Δ*G*_cavity_ for the ligands, free in solution with their structures fixed as observed in the respective complexes, are shown in Table 2 ▸.

Consistent with the extra methyl group, the cavity term for MZM is less favorable than that for AZM by ∼1.5 kcal mol^−1^. This means that when free in solution MZM must dispel more water to accommodate its additional methyl group and for this it must pay a higher free-energy penalty than does AZM. Although this free-energy penalty is partly offset by the dispersion term, the solvation of AZM is still slightly favored over that of MZM.

**Figure 4:**
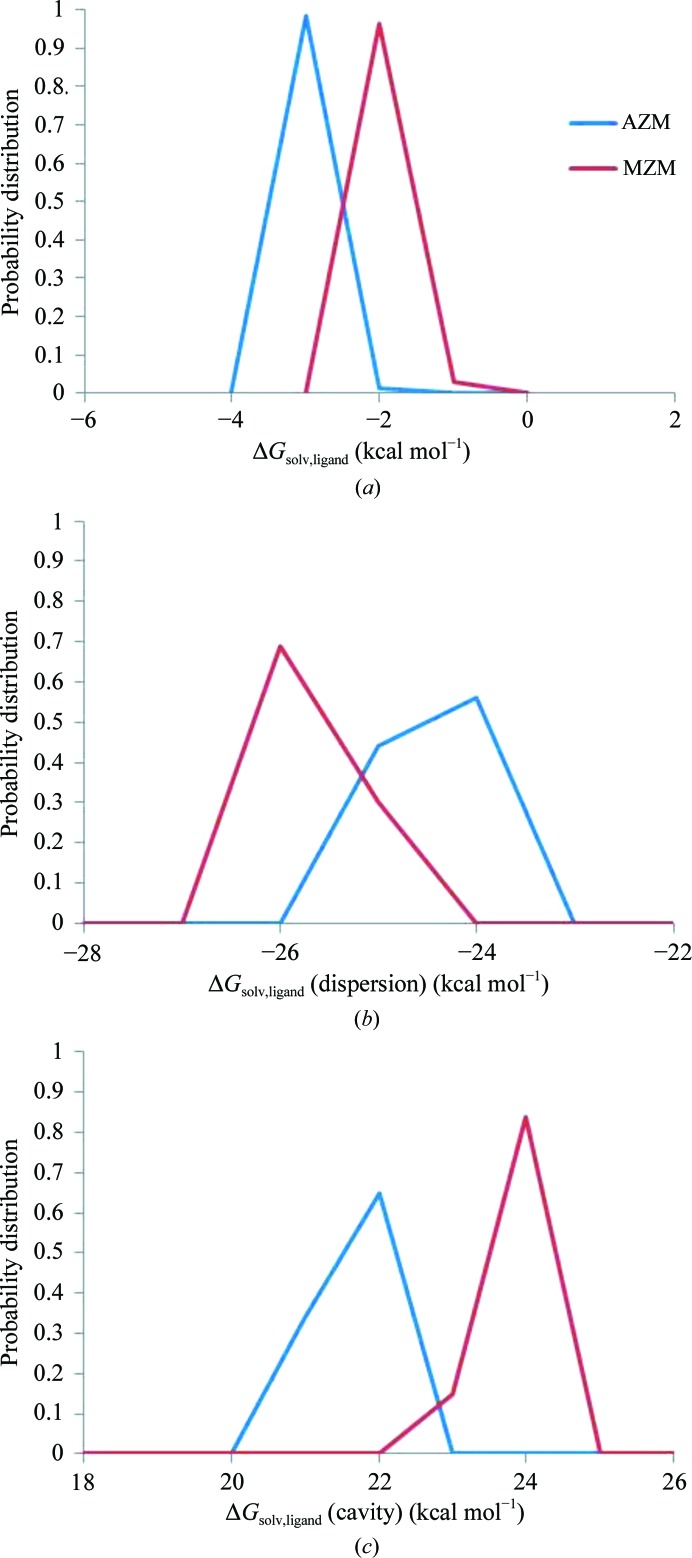
(*a*) Nanopolar solvation energy of AZM and MZM (Δ*G*_solv,ligand_). (*b*, *c*) The contribution of dispersion (*b*) and cavity (*c*) to Δ*G*_solv,ligand_.

In a further set of calculations, the flexibility of the ligands in solution was taken into account through the use of molecular-dynamics simulations. The solvation energies of the complexes and enzyme were also calculated, but the solvation energies of the ligands in solution are sufficient to rationalize the differences in the structure of the ordered waters in the binding pockets for the two complexes in this study. The solvation energies of the ligands alone were decomposed into contributions from attractive dispersion and repulsive cavity contributions. These calculations provided a distribution of the values of the solvation energies as the free ligand configurations fluctuate in solution. As with the static structures from the neutron crystallography experiments, Δ*G*_solv_ is still more favorable for AZM than MZM (Fig. 4 ▸). This additional set of calculations shows that MZM has a more energetically favorable contribution from dispersion while retaining an increased ability to dispel water molecules relative to AZM (Fig. 4 ▸). Fig. 4 ▸ emphasizes the need to sample the neighborhood immediately around the pose found in the crystal structure. If only the pose in the crystal structure had been analyzed (Table 2 ▸), it would not have been found that MZM has a more energetically favorable contribution from dispersion than AZM.

These calculations indicate that the loss of binding energy of MZM accompanying excess water-molecule expulsion relative to AZM will be largely offset by the favorable contribution from dispersion for MZM. While the model is simple, the analysis illustrates how factors that are not always apparent from the examination of bound crystal structures (especially those from data collected at cryotemperatures) might influence ligand binding/water behavior.

## 4. Conclusion   

The relative importance of hydrogen bonds and hydrophobic interactions has long been debated with regard to their relative importance in ligand (drug) binding. In the case of MZM, hydrophobic forces perhaps compensate for the loss of an extensive hydrogen-bonding network. This balancing of entropies and hydrogen-bonding forces could well be argued as the reason behind the similar inhibition constants (10 n*M*) of AZM and MZM (Maren *et al.*, 1993 ▸). It should be noted that the ostensible displacement of four more waters by MZM compared with AZM is in fact a displacement of one water and disorder of three other waters compared with the X-ray cryo structure of the hCA II–MZM complex.

In summary, we have determined the RT X-ray/neutron structure of hCA II in complex with MZM (PDB entry 5c8i). These X-ray/neutron RT structures, X-ray cryo structures and molecular-dynamics calculations have allowed the first detailed comparative study of two clinical drugs (the other being AZM) in complex with their biological target. The study provides new insights into the hydrogen-bonding changes that take place within the active site of an enzyme upon drug binding, including changes in the orientation of H atoms of residues and waters, and the observed displacement of water.

## Supplementary Material

PDB reference: hCA II, complex with methazolamide, 5c8i

## Figures and Tables

**Table 1:** Crystallographic details of the jointly refined (X-ray/neutron) hCA II–MZM structure Values in parentheses are for the highest resolution bin.

PDB code	5c8i
Data-collection statistics	
Space group	*P*2_1_
Unit-cell parameters (Å, °)	*a* = 42.9, *b* = 41.7, *c* = 73.0, α = 90.0, β = 104.6, γ = 90.0
Reflections	
Measured	39068
Unique	10417
Resolution (Å)	41.5–2.2 (2.3–2.2)
*R*_merge_ †	0.17 (0.08)
〈*I*/σ(*I*)〉	4.1 (2.6)
Completeness (%)	80.6 (66.0)
Multiplicity	3.8 (3.5)
Data-rejection criteria	No observation and |*F*| = 0
X-ray refinement	
*R*_cryst_ ‡/*R*_free_ § (%)	16.0/17.8
R.m.s.d.	
Bond lengths (Å)	0.006
Bond angles (°)	1.17
Joint XN refinement	
Resolution, neutron (Å)	40.0–2.2
Resolution, X-ray (Å)	40.0–1.5
No. of reflections (neutron)	9193
No. of reflections (X-ray)	31814
*R*_cryst_/*R*_free_ (neutron)	0.22/0.27
*R*_cryst_/*R*_free_ (X-ray)	0.20/0.22
No. of atoms	
Protein	4056
Ligand	21
Water	231
Average *B* factors (Å^2^)	
Protein	20.4
Ligand	17.0
Water	36.9
R.m.s.d.	
Bond lengths (Å)	0.006
Bond angles (°)	0.99

† *R*_merge_ = 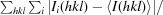 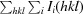 × 100.

‡ *R*_cryst_ = 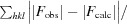 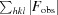 × 100.

§ *R*_free_ is calculated in the same manner as *R*_cryst_ except that it uses 5% of the reflection data that were omitted from refinement.

**Table 2:** Cavity and dispersion terms for AZM and MZM in free solution Energies are in kcal mol^−1^.

	Δ*G*_solv_	Δ*G*_cavity_	Δ*G*_dispersion_
AZM	−3.0	21.9	−24.9
MZM	−2.2	23.5	−25.8
